# Semantic Similarity Search Approach to Extract Exemplars of Stigmatizing and Positive Language in Obstetric Clinical Notes: Exploratory Study

**DOI:** 10.2196/85088

**Published:** 2026-09-22

**Authors:** Jihye Kim Scroggins, Ismael Ibrahim Hulchafo, Veronica Barcelona, Anahita Davoudi, Hans Moen, Sarah Harkins, Danielle Scharp, Kenrick Cato, Michele Tadiello, Maxim Topaz

**Affiliations:** 1 School of Nursing, University of North Carolina at Chapel Hill, 120 Medical Drive, Chapel Hill, NC, 27514, United States, 1 (919) 966-4260; 2 School of Nursing, Columbia University, New York, NY, United States; 3 VNS Health, New York, NY, United States; 4 Department of Computer Science, Aalto University, Espoo, Finland; 5 Icahn School of Medicine at Mount Sinai, New York, NY, United States; 6 School of Nursing, University of Pennsylvania, Philadelphia, PA, United States; 7 Center for Community-Engaged Health Informatics and Data Science, Columbia University Irving Medical Center, New York, NY, United States

**Keywords:** natural language processing, electronic health records, stigmatizing language, health communication, bias

## Abstract

**Background:**

Natural language processing can extract meaningful information from clinical notes. However, human annotation is time-consuming and costly, and scarce data poses a challenge.

**Objective:**

This study aimed to explore a semantic similarity search approach to extract exemplars of stigmatizing and positive language in obstetric clinical notes.

**Methods:**

We used electronic health record data from labor and birth admissions at 2 hospitals in the United States from 2017 to 2019. We used a semantic similarity search approach, which used 200 randomly selected true exemplars, stratified by language categories, as queries to search for similar exemplar candidates. We extracted the top 5 candidates with the highest cosine similarities, which were assessed for accuracy.

**Results:**

We retrieved 1000 candidates. An average precision of 0.69 was achieved when candidates with cosine similarity thresholds of 0.75 or higher were included, at which point 68.8% (64/93) of exemplar candidates accurately represented true cases. At the 0.75 threshold, the proportion of true cases was higher for preferred language (41/56, 73.2%) and unilateral/authoritarian decisions (5/7, 71.4%). The proportion of true cases was lower for difficult patients (2/7, 28.6%) and marginalized identities (3/9, 33.3%).

**Conclusions:**

The semantic similarity search approach shows promise in efficiently extracting exemplars while reducing the annotation burden, laying the groundwork for future applications in other domains.

## Introduction

Natural language processing (NLP) can efficiently and accurately extract meaningful information from large text data, such as clinical notes within electronic health records (EHRs) [1,2]. One significant challenge is obtaining sufficient human-annotated data to train NLP models for specific tasks and datasets. Human annotation is labor-intensive and costly, requiring extensive effort to review and label data for exemplars representing specific concepts or categories within the text. For example, annotating 50 discharge summaries can take approximately 80 hours for a team of several clinical experts; this time grows exponentially for datasets with a low prevalence of the target information [3]. Given the substantial time and manpower needed for human annotation, it is common to encounter limited human-labeled data for NLP model development [4].

Prior NLP research has focused on reducing annotation burden through approaches such as active learning and data augmentation. Traditional active learning, most commonly using uncertainty-based querying, supports annotation by iteratively selecting unlabeled cases for which the model has the highest uncertainty (lowest confidence) and prioritizing these cases for human review [5]. However, uncertainty-based querying may be less effective for low-prevalence or rare concepts, which can be under-selected when models exhibit high confidence in assigning majority-class labels; for example, rare positive cases may be confidently misclassified as negative and therefore not selected for human review [6,7]. Data augmentation, including synonym replacement, paraphrasing, and more recently, synthetic data generation using large language models, has also been used to expand training datasets [8,9]. However, these approaches may unintentionally introduce noise or alter subtle linguistic or contextual cues essential for capturing nuanced context-dependent concepts [10,11]. For example, human evaluation of synthetic data quality in the context of stigmatizing language has shown that synthetic exemplars exhibited limited clinical realism [12].

Semantic similarity search offers a complementary strategy for expanding training data by efficiently extracting additional exemplars that are semantically similar to existing human-annotated exemplars. Semantic similarity refers to the degree to which 2 pieces of text share similar meanings based on their linguistic content and context [13]. Semantic similarity has been widely studied. Early approaches focused on document-level similarity based on lexical and distributional overlap [14]. More recent work has focused on sentence-level similarity using neural embeddings, which enable more nuanced comparisons across short text spans [15]. In the clinical domain, prior work has largely focused on developing and evaluating models for estimating similarity between pairs of clinical sentences [16,17]. These studies typically use trained models to predict similarity scores between sentence pairs and evaluate how closely these predictions align with human ratings [16,17]. Semantic similarity has also been used in information retrieval and retrieval-augmented generation systems to retrieve clinically relevant texts, passages, or documents [18,19]. These approaches are primarily used for downstream tasks such as question answering and clinical decision support [20]. In contrast, the use of semantic similarity search to support annotation workflows, specifically for retrieving additional exemplars, remains relatively limited.

In the current study, we applied semantic similarity search to support annotation by using human-annotated true exemplars as queries to retrieve additional exemplar candidates. This approach has the potential to reduce the human labor associated with reviewing and labeling new text data from scratch. Unlike traditional active learning, this approach directly retrieves semantically similar exemplar candidates to human-annotated true exemplars, without relying on iterative model training or uncertainty-based querying. This strategy may be particularly useful for identifying rare concepts, as it can retrieve semantically similar text even when such cases are infrequent, allowing expansion of sparse categories. In addition, by using true exemplars as queries, this approach can preserve real-world language and subtle contextual features that may not be well captured by synthetic data.

Specifically, we explored the application of this approach in the context of identifying stigmatizing and positive language categories in obstetric clinical notes. Stigmatizing language can convey implicit or explicit bias [21], which can negatively influence patient-clinician relationships and satisfaction in health care [22,23]. Identifying and mitigating stigmatizing language is essential for providing respectful and unbiased care for perinatal populations affected by significant health disparities [24]. The prevalence of stigmatizing language can be as low as 1% to 2% for certain language categories or patient populations [25,26]. This presents challenges for manual annotation and obtaining sufficient data for optimal NLP model training. Additionally, identifying positive language that respects patients’ views and autonomy is important to promote the use of strength-based language in clinical documentation [27]. Our efforts to identify stigmatizing and positive language revealed the need for an efficient approach to extract additional exemplars with limited resources [28]. This study aimed to evaluate semantic similarity search as a more efficient approach for identifying additional exemplars of stigmatizing and positive language in obstetric clinical notes. We assessed retrieval accuracy through human evaluation and precision metrics.

## Methods

### Study Data

We used EHR data from patients at >20 weeks’ gestation admitted for labor and birth at 2 urban hospitals in the Northeast United States from 2017 to 2019. All clinical notes in the EHR during the inpatient hospital stay were eligible. Note types with limited clinician narrative text describing patient assessments or impressions, such as medication orders, transfer notes, and template-based statements about procedures or operations, were excluded. Seven note types were used in the current study: obstetric postpartum note, obstetric admission note, obstetric triage note, anesthesia resident note, miscellaneous nursing note, social work initial assessment, and initial nutrition assessment.

### Ethical Considerations

We received institutional review board (IRB) approval from Columbia University Medical Center (AAAT9870) for this study under expedited review, category 5: research involving materials (data, documents, records, or specimens) that have been collected or will be collected solely for nonresearch purposes, such as medical treatment or diagnosis. A waiver of informed consent was granted by the IRB because the research involved no more than minimal risk, did not adversely affect the rights and welfare of human subjects, could not be carried out without the waiver, and the study could not be completed without using identifiable private information from patients discharged from the study hospitals. To protect privacy and confidentiality, study data were stored on a secure server following institutional data security guidelines, with access restricted to authorized study personnel with a direct need for data management and analysis. All study personnel completed IRB-required training in the responsible conduct of research and protection of human subjects. No compensation was provided because this study involved secondary use of data without direct participant involvement, interaction, or recruitment.

### Semantic Similarity Search Approach

#### Approach Overview

We explored using semantic similarity search to expand the initial human-annotated dataset in a less manually intensive way. This approach uses initial human-annotated exemplars (ie, *true exemplars*) as queries to search for similar exemplars (ie, *exemplar candidates*). Then, human experts can perform a relatively faster review of the exemplar candidates to determine the accuracy.

#### Language Categories

Stigmatizing and positive language categories are presented in Table 1. The categories and operational definitions were developed through iterative qualitative analysis [26,29] and informed by prior work on stigmatizing language [30]. A multidisciplinary team of experts, including obstetrics and gynecology physicians, nurses, and nonclinical researchers, contributed diverse perspectives to the development of these categories. These efforts were intended to enhance the rigor of the operational definitions.

**Table 1.** Stigmatizing and positive language categories.

Language category	Definition	Examples	True exemplars (N=200), n
Stigmatizing language			
Difficult patient	Nonadherence or noncompliance with plan of care, refusal of care, referrals, or services.	“missed nutrition appt again says she doesn’t want to go.” “exam unchanged however now c/o [complains of] ctx [contraction] pain.” “poor compliance with antenatal visits.”	28
Unilateral/ authoritarian decisions	Language that supports clinician’s authority over patients. Upholds hierarchy centering clinician, not patient.	“cervix unfavorable therefore will start induction with Cytotec.” “SW [social worker] has advised pt [patient] that if there continues to be yelling in room, ACS [adult and child services] will need to be contacted.” “also very upset about persistent shaking in her UE [upper extremities]. Explained to patient multiple times that shaking is normal and a result of hormones and the anesthesia.”	14
Power/privilege	Describes power and privilege identities related to psychological or social-ecological status.	“pt [patient] reports having a nurturing marriage with FOB [father of the baby] who works as a Lawyer.” “private pt [patient] of mine at 39+6 wks [weeks] with multiple episodes of emesis this AM.” “Caucasian female. pleasantly engaged in conversation.”	17
Marginalized identities	Documentation of social and behavioral risk factors in narrative that could contribute to marginalization. Restating for emphasis that is already in the checklist/form data or unnecessary patient descriptor (eg, “toxic habits,” “financially supports self,” “teen mother,” “late registrant,” and “obesity”).	“social disarray, estranged from family with FOB [father of the baby] not involved.” “Dominican female. Initial psychosocial assessment due to teen pregnancy late registrant.” “Patient is a 32yo [year old] Dominican unmarried unemployed female.”	74
Questioning patient credibility	Disbelief in patient’s report of health and social history or status.	“unsure if patient telling the truth.” “pt [patient] denied any other DV [domestic violence] incidents and was adamant that relationship with spouse was healthy.” “Pt [patient] does not think she has gestational diabetes she states she ate very sweet rice (a dish from her country) the day before the glucose challenge which is what she believes this is the reason for the high value states it has never been high before.”	11
Disapproval	Behaviors of patient not in alignment with health care provider expectations.	“FOB [father of baby] is 23 yo [year old] unemployed and not as involved as he should be.” “Pt [patient] states she tried to obtain f/u [follow-up] appt [appointment]. but did not get a call back. Advised she should have gone to the clinic to straighten things out- in the future to take initiative-importance stressed as well as appts [appointments] for her newborn.” “PP [postpartum] BCM [bridge contraceptive method]- pt states she prefers to use condoms will continue to readdress.”	6
Structural/interprofessional hierarchy	Notes stating issues in care are attributed to another clinician, often nurses. Notes stating that care was not delivered in a timely manner due to staffing or other structural issues.	“Nurse states IV [intravenous] ‘infiltrated,’ but it was fine.” “Unable to staff nursing for placement of epidural in triage. Requested that charge nurse please inform anesthesiology team about when there is enough nursing staff.” “Patient continues to wait in triage until L D [Labor & Delivery] bed available.”	2
Positive language			
Preferred language	Preferred words that can convey patient’s point of view respectively and objectively (eg, endorses, reports, or states).	“patient desires to ambulate and encourage natural labor.” “patient states she feels some pain on right side.” “states she has had irregular ctx [contractions] starting 24 hours ago. endorses FM [fetal movement]. Endorses N/V [nausea/vomiting]”	44
Autonomy for birth	Notes that indicate patient exercising autonomy around birth and plan of care.	“Pt [patient] declines epidural at this time- trying to deliver without analgesia.” “will give patient the option to have continuous monitoring overnight and give her the opportunity to go into spontaneous labor with plans for c-section tomorrow if she does not progress.” “low risk screening declined diagnostic. She was given the option of admission with possible augmentation of labor vs discharge home to labor. Plan was made for admission.”	4

#### Initial Human Annotation

To generate the initial human-annotated dataset, 4 researchers with expertise in qualitative research and/or nursing independently annotated clinical notes following an established codebook (see Table S4 in Multimedia Appendix 1). The codebook was developed through iterative inductive-deductive content analysis [26]. All annotators were trained prior to participating in annotations, which included review of the codebook, discussion of example phrases and sentences, and coding demonstrations. Annotators manually labeled true exemplars from a total of 1771 clinical notes, which typically spanned 1 to 3 sentences. To ensure reliability, 2 annotators reviewed and annotated the same clinical notes. We resolved disagreements through iterative discussions among the annotators. The initial agreement among the annotators across the 1771 clinical notes was fair (Cohen κ=0.4, agreement rate=72%), which reflects the inherently nuanced nature of language use and subjectivity involved with interpreting complex and nuanced language [31]. We spent extensive effort and time discussing any discrepancies among annotators to reach a consensus and ensure the quality and accuracy of the final annotated dataset [31].

#### Sentence-Transformer Model

We used a sentence-transformer model, “multi-qa-distilbert-cos-v1,” from the Hugging Face Model Hub [15,32] to search for additional exemplar candidates that were semantically similar to the set of true exemplars. This model is specifically designed for semantic similarity search and retrieval tasks using a contrastive learning objective to generate sentence-level embeddings that enable comparison of a given sentence with other sentences that are most closely related in meaning [32]. This design enables efficient, out-of-the-box application of the model for similarity-based retrieval tasks, making it well suited for the current task. Although this model is not trained on clinical text, it is trained on large-scale, question-answer-style data from diverse sources [32], which contributes to capturing contextual meaning across varied linguistic expressions. This general-domain training may be helpful for identifying broader sociolinguistic patterns and contextual features in clinical text that can extend beyond clinical language. Prior work suggests that general-domain embedding models can perform well for semantic similarity tasks in clinical text, although performance may vary across specific models and tasks [33].

Exemplar candidates were selected from clinical notes not used in the initial human annotation. We preprocessed the clinical notes by converting the free-text portions into a format similar to the true exemplars (single, double, and triple consecutive sentences). Sentence tokenization was performed first using natural language toolkit’s “sent_tokenize,” which identifies sentence boundaries based on learned punctuation and linguistic patterns rather than simple rule-based splitting. After tokenization, sliding windows of 1-, 2-, and 3-sentence spans were generated by advancing one sentence at a time. Duplicate candidates were then detected using a 6-word prefix and removed in 2 stages: first keeping only the earliest instance of each prefix, and then alternately dropping any remaining repeats to ensure only 1 representative remained. No normalization of casing, numbers, or abbreviations was applied. All text was embedded in its original form to preserve real-world clinical text. Examples of synthetic, free-text note snippets can be found in Table S3 in Multimedia Appendix 1.

We used the sentence-transformer model to encode true exemplars into fixed-size vectorized embeddings [15]. Then, true exemplars were used as queries to search and recommend new exemplars that were likely candidates from the unused clinical note datasets. We calculated semantic similarity between the true exemplars and exemplar candidates using the cosine similarity applied to their associated embedding representations. The Facebook AI similarity search library was used to perform the similarity searches [34]. We stratified and randomly selected 200 true exemplars across language categories to be used as queries. Stratification was conducted by language category to preserve each category’s natural prevalence in the initial human-annotated dataset. As a result, less frequent categories contributed fewer exemplars (Table 1). For each of the 200 true exemplars, the top 5 similar exemplar candidates with the highest cosine similarities were retrieved (N=1000 exemplar candidates) for human review. Pseudocode blocks for the semantic similarity search pipeline employed in this study are provided in Multimedia Appendix 2.

### Examining Accuracy of Exemplar Candidates

Four researchers who performed the initial annotation independently reviewed and assessed the exemplar candidates. Reviewers labeled the exemplar candidate as a “match” if it accurately represented the corresponding language category and as “not a match” if it did not. We resolved disagreements through iterative discussions among reviewers to reach consensus. We calculated the frequency and percentage of matched and nonmatched cases as true positives and false positives. We examined how precision changes when different cosine similarity boundaries were used to inform optimizing this approach for future research and applications. We calculated and reported average precision and corresponding 95% Wilson CIs across different cosine similarity thresholds, ranging from 0.5 to 0.95 in increments of 0.05. To our knowledge, there is no universally accepted precision cutoff for information retrieval tasks to support annotation workflows. Information retrieval literature supports that performance should be evaluated in relation to the intended use and context of each task, as performance requirements may vary across applications [35]. Previous research on annotation support often focuses on reducing annotation burden while maintaining annotation quality [36,37]. In a recent study on automated annotation support, the authors noted that precision exceeding 0.7 was considered “strong performance” for their task [38]. Given the exploratory nature of this study and its intended use to support annotation workflows, we selected precision ≥0.70 as an acceptable, pragmatic point to balance accuracy with meaningful efficiency gains in reducing annotation burden. At this precision point, most exemplar candidates would represent true cases while allowing a manageable proportion of false positives.

## Results

**Figure 1.**
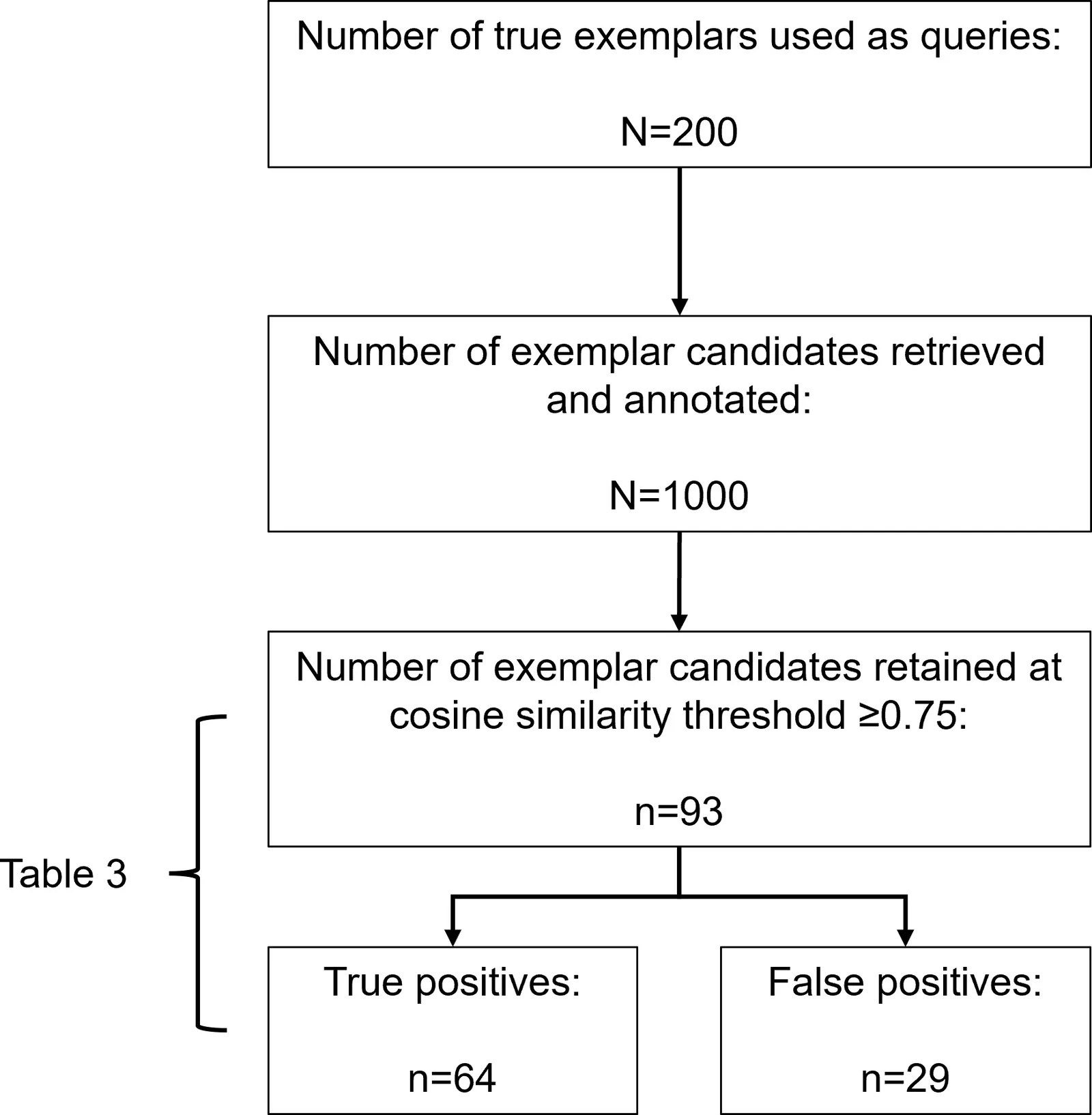
Flow diagram summarizing the number of true exemplars or exemplar candidates in the analysis and results.

A total of 1000 exemplar candidates were retrieved using the semantic similarity search approach (Figure 1). These exemplar candidates were derived from 443 clinical notes of 403 patients. The number of exemplar candidates by note type is provided in Table 2. Among the 1000 exemplar candidates retrieved, most were derived from obstetric admission notes (n=295, 29.5%), followed by obstetric postpartum notes (n=202, 20.2%) and miscellaneous nursing notes (n=172, 17.2%). Human annotators independently reviewed all 1000 exemplar candidates retrieved for accuracy. The interrater reliability among the annotators was good (Cohen κ=0.71, 95% CI 0.67‐0.75) across 1000 exemplar candidates.

**Table 2.** Number of exemplar candidates by note type.

Note type	Exemplar candidates^a^, n (%)
Obstetric admission note	295 (29.5)
Obstetric postpartum note	202 (20.2)
Miscellaneous nursing note	172 (17.2)
Anesthesia resident note	137 (13.7)
Obstetric triage note	106 (10.6)
Social work initial assessment	51 (5.1)
Initial nutrition assessment	37 (3.7)
Total exemplar candidates retrieved	1000 (100)

a The total number of exemplar candidates retrieved was used as the denominator to calculate the percentages.

**Figure 2.**
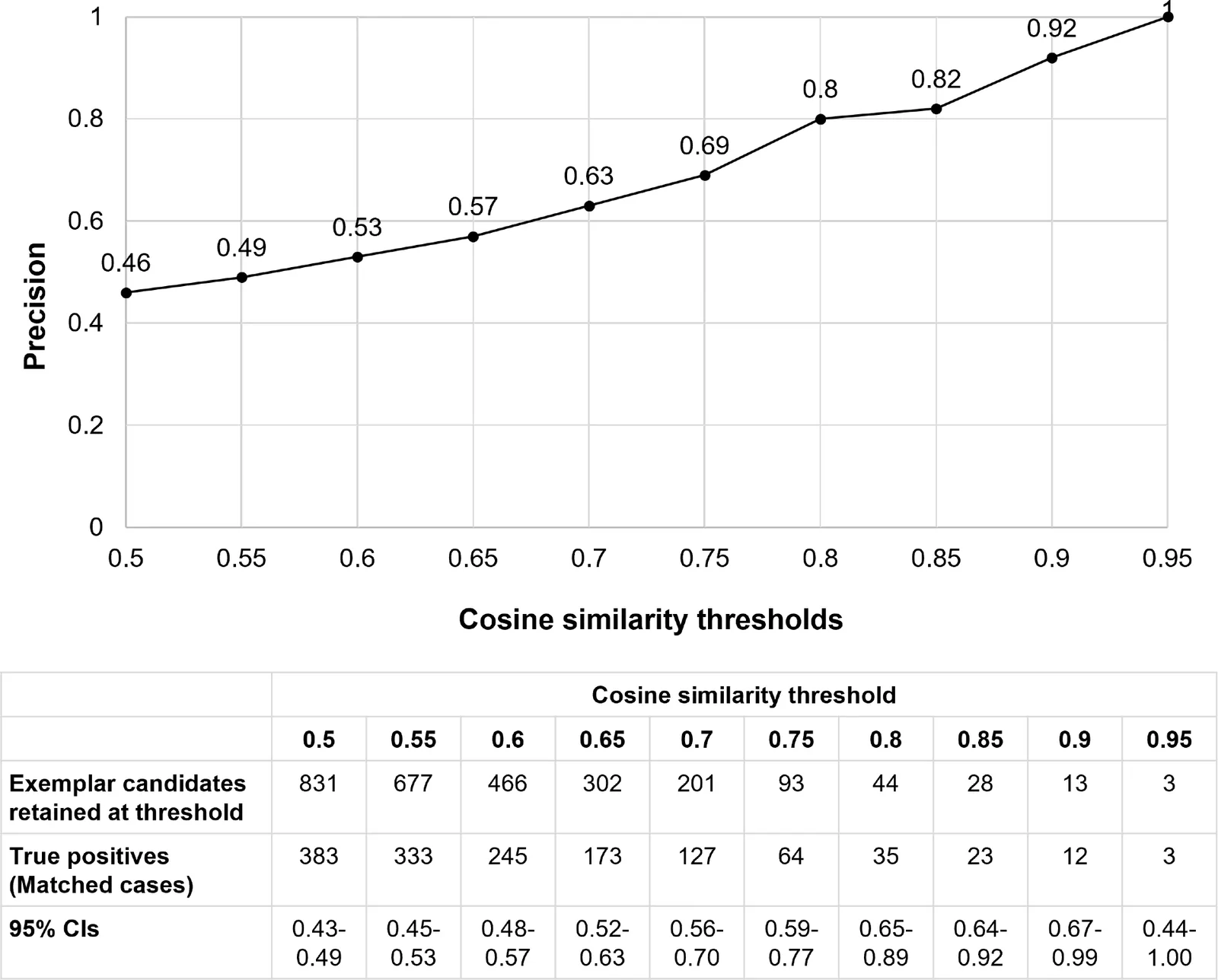
Line graph illustrating the average precision at different cosine similarity thresholds with corresponding values for the total number of exemplar candidates retained at each threshold, the number of true positives, and 95% CIs. The total number of exemplar candidates retained at each threshold was used as the denominator to calculate the precision.

We examined how the average precision of all language categories changed when including exemplar candidates at different cosine similarity thresholds. As shown in Figure 2, average precision increased with higher cosine similarity thresholds. The number of exemplar candidates retained decreased with higher cosine similarity thresholds. Average precision was highest at 1 (95% CI 0.44‐1.00) when including exemplar candidates with cosine similarity ≥0.95, at which point 3 of 3 exemplar candidates were true cases. Average precision was lowest at 0.46 (95% CI 0.43‐0.49) when including exemplar candidates with cosine similarity ≥0.5, at which point 383 of 831 exemplar candidates were true cases. Acceptable average precision (≥0.70) was achieved when including exemplar candidates with a cosine similarity of at least 0.75.

A total of 93 exemplar candidates had a cosine similarity of at least 0.75. Among those, 64 (68.8%) accurately represented the true cases across stigmatizing and positive language categories (Table 3). In specific language categories, 100% (5/5) of exemplar candidates for autonomy for birth, 73.2% (41/56) for preferred language, and 71.4% (5/7) for unilateral/authoritarian decisions represented the true cases. While the proportions of matched cases were high for power/privilege, questioning patient credibility, and disapproval, the number of retrieved exemplar candidates for these categories was small at the 0.75 cosine similarity threshold (eg, 1 of 1 exemplar candidate was a true case for power/privilege and disapproval, respectively). In contrast, 28.6% (2/7) of exemplar candidates for difficult patient and 33.3% (3/9) of exemplar candidates for marginalized identities represented the true cases, with lower proportions of matched cases than other language categories at the 0.75 cosine similarity threshold.

**Table 3.** Proportion of matched cases by language category at a cosine similarity threshold of 0.75.

Language category	Exemplar candidates, N^a^	True positives (matched cases), n (%)	False positives (nonmatched cases), n (%)
Preferred language	56	41 (73.2)	15 (26.8)
Autonomy for birth	5	5 (100)	0 (0)
Difficult patient	7	2 (28.6)	5 (71.4)
Unilateral/authoritarian decisions	7	5 (71.4)	2 (28.6)
Power/privilege	1	1 (100)	0 (0)
Structural/interprofessional hierarchy	4	3 (75)	1 (25)
Marginalized identities	9	3 (33.3)	6 (66.7)
Questioning patient credibility	3	3 (100)	0 (0)
Disapproval	1	1 (100)	0 (0)
Total	93	64 (68.8)	29 (31.2)

a The total N column was used as the denominator to calculate the proportion of matched and nonmatched cases.

Detailed results for precision at different cosine similarity thresholds by specific language categories and corresponding CIs are reported in Tables S1 and S2 in Multimedia Appendix 1. Of note, several precision values were based on small numbers of exemplar candidates, particularly at higher thresholds, and are statistically unstable for these sparse categories. For categories with relatively larger numbers of retained candidates, such as preferred language, acceptable precision (≥0.70) was observed at a cosine similarity of 0.70, where 74 of 105 exemplar candidates represented true cases. False positives most commonly occurred in exemplars containing more complex social or contextual information. For example, exemplars describing objective social circumstances were misclassified as stigmatizing despite the absence of judgmental language (eg, false positive in the marginalized identities category: “Patient may want to be discharged to home tonight. She lives nearby and will be able to visit infant”). In other cases, exemplars containing stigmatizing language were misclassified into incorrect categories. For example, “Patient stated she doesn’t have any help in the country and FOB [father of baby] is in DR [Dominican Republic]. Patient needs social worker, will continue to monitor...” was incorrectly classified into the power/privilege category.

## Discussion

### Principal Findings

We explored a semantic similarity search approach to identify additional exemplars from obstetric clinical notes with less manual human effort. We found that we can increase accuracy by adjusting cosine similarity boundaries when selecting exemplar candidates. The average precision increased with higher cosine similarity thresholds, achieving an acceptable average precision ≥0.70 when exemplar candidates with a cosine similarity of 0.75 or higher were included. On average, 68.8% (64/93) of exemplar candidates accurately represented true cases across various language categories at the 0.75 cosine similarity threshold. These thresholds should be guided by each use case in future research and application. Lower thresholds may be useful for exploratory analyses, where broader retrieval is desired to examine patterns of expressions and language use. Lower thresholds may also be appropriate when maximum coverage is prioritized over precision, such as for rarely occurring cases, where missing potential matches would be more consequential than reviewing additional false positives. In contrast, higher thresholds may be suitable when precision should be prioritized, such as using retrieved texts for direct application in clinical settings, including quality review or clinical documentation audit to identify potential stigmatizing language. Higher thresholds may also be useful with large datasets, where even a strict precision cutoff can yield a sufficient number of exemplar candidates, whereas the same threshold applied to smaller datasets may result in too few retrieved exemplars to be useful.

It is important to note that the reported accuracy in our study reflects only positive retrieval quality, measured by precision, rather than the overall effectiveness or completeness of the retrieval approach. Given the exploratory nature of the study, we did not annotate nonretrieved texts to calculate more comprehensive evaluation metrics, including recall and *F*_1_-scores. As such, our findings do not provide information regarding the extent to which the approach identified all relevant exemplars within the dataset. For example, it remains unknown how many relevant exemplars may not have been retrieved, limiting the ability to assess the completeness of retrieval. In addition, the number of exemplar candidates retained decreased substantially across language categories as cosine similarity thresholds increased (detailed category-specific results in Multimedia Appendix 1). As such, the current study may be underpowered to support reliable category-level conclusions. When few exemplar candidates were retained, high precision values should not be misinterpreted as strong performance, as they are statistically unstable and reflect limited sample size. At lower cosine thresholds, where more candidates were retained, precision varied across language categories. Although these should be interpreted cautiously, they may reflect different language complexity. For example, at the 0.5 cosine similarity threshold, higher precision was observed for preferred language and autonomy for birth compared to other categories. These 2 categories were more straightforward with well-defined, identifiable keywords (eg, “desires natural birth,” “reports pain”). Conversely, we observed lower precision for marginalized identities, questioning patient credibility, and disapproval categories at the 0.5 cosine similarity threshold. These categories were more nuanced and context-dependent. For example, there was a subtle undertone of disapproving the patient’s choice of birth control in the following: “postpartum birth control method-patient states that she prefers to use condoms and will continue to readdress.”

Accurately identifying such nuances requires a deeper understanding and interpretation of contextual meanings and subtleties, which may be challenging for semantic similarity search. Semantic similarity search primarily relies on vector-based representations of text, including static embeddings like Word2Vec and GloVe [13,39] and contextual embeddings like bidirectional encoder representations from transformers (BERT) [40]. Static embeddings can effectively capture general semantic relationships [13,39] but can be limited in detecting context-specific nuances and meanings [41]. Although contextual embeddings, such as those generated by the sentence-transformer model used in the current study, can better capture contextual information, they may still be challenged by complex and subtle nuances [42]. Finally, when suitable training data are available, sentence-transformer models could be further improved through data- and task-specific training by fine-tuning.

While semantic similarity has been previously used for tasks such as information retrieval, its application to support annotation workflows, specifically for retrieving additional exemplars, remains relatively limited. The current study extends this line of work by applying semantic similarity search to more efficiently expand annotated datasets. This approach is particularly useful for low-prevalence concepts, where traditional active learning strategies may be less effective. In contrast to data augmentation strategies, such as synthetic data generation, it can preserve naturally occurring language while enabling targeted expansion of context-dependent concepts. Furthermore, while prior retrieval-based approaches have used similarity to identify relevant text for tasks such as information retrieval or retrieval-augmented generation, the effectiveness of these approaches is typically evaluated based on automated metrics or downstream model performance, rather than human validation [18,19]. In contrast, we conducted human evaluation of retrieved exemplar candidates and examined precision across cosine similarity thresholds, providing insight into the utility of this approach as an annotation support strategy. Our findings highlight how cosine similarity thresholds influence precision, offering practical guidance for applying this approach in settings where annotated data are sparse.

Although our findings are based on the context of stigmatizing and positive language in obstetric clinical notes, which may limit the generalizability to other specialized datasets or domains, semantic similarity search itself is not inherently domain-specific. Because this approach operates by comparing a given sentence with other sentences to identify those most closely related in meaning, it is not dependent on or limited to specific models but can be incorporated into existing clinical NLP pipelines as a data augmentation or annotation-support step. For example, retrieved exemplar candidates may be used to efficiently expand training datasets prior to NLP model development or to support targeted human review in resource-constrained settings. Prior research has demonstrated the practical benefits of targeted text selection using active learning to support annotation workflows. For example, active learning approaches have been shown to reduce annotation time by approximately 20% to 35% for clinically explicit concepts, such as medical problems and tests, by prioritizing informative texts for manual annotation [43,44]. More recently, active learning has also been shown to reduce other forms of annotation burden. For example, Nachtegael et al [45] used active learning to select unlabeled texts for manual annotation. They found that models trained on selectively annotated datasets achieved performance comparable to models trained on fully labeled datasets while reducing the amount of labeled data required by 6% to 38% [45]. Although these studies employed different active learning approaches than the current study, they indicate that strategically selecting texts for manual review can reduce annotation burden.

### Limitations

This study has some limitations. First, the effectiveness of semantic similarity search depends on the quality of the initial human-annotated dataset. If true exemplars do not accurately reflect positive cases, the extracted candidates are also likely to be inaccurate. The relatively modest initial agreement among annotators (Cohen κ=0.4) highlights the subjective nature of identifying stigmatizing language. Consequently, some retrieved exemplars may have been classified differently among annotators, which could have influenced downstream retrieval accuracy. Additionally, if true exemplars do not represent diverse language categories, the extracted candidates may not be comprehensive. Second, while semantic similarity search reduced the labor required for annotation, it does not eliminate the need for human review. Due to the risk of false positives, a rapid human review of the retrieved candidates remains essential to validate the accuracy. Third, our evaluation was limited to precision to prioritize annotation efficiency. In our approach, the model retrieved exemplars predicted to be positive matches to the true exemplars. We did not annotate nonretrieved texts to identify false negatives, which is required to calculate recall. As such, the expanded dataset should be useful for augmenting existing annotated datasets with positive cases, rather than providing exhaustive or population-representative datasets. Fourth, although language categories and operational definitions were developed through qualitative analysis and informed by prior research, several categories remain inherently subjective and context-dependent. The operational definitions for these categories may remain somewhat ambiguous in practice, which can introduce variability in interpretation even among trained annotators. This reflects broader challenges in objectively operationalizing stigmatizing language in clinical notes, and findings should be considered given this inherent measurement limitation. While all discrepancies were resolved through consensus to establish the final annotated dataset, some degree of uncertainty may remain. As such, the retrieved exemplars may reflect alignment with the study-specific definitions and exemplars rather than universally accepted definitions. Fifth, we used a model trained on general-domain data because it is designed for similarity-based retrieval tasks [32], enabling its direct and efficient application in the current study without additional modifications. In addition, stigmatizing and positive language often requires capturing broader sociolinguistic expressions and context beyond clinical language, for which training on diverse data sources may be helpful. However, clinical language often includes specialized terminology, abbreviations, and domain-specific cues, which may not be fully captured by general-domain embedding models. As such, semantic similarity estimates may be less accurate for certain clinical expressions. Domain-specific models (eg, ClinicalBERT or BioClinicalBERT) are trained on clinical text and may provide better representations of clinical terminology and context [46]. However, these models are not specifically designed or explicitly optimized for sentence-level similarity search or retrieval tasks. Thus, additional methodological adaptations, such as deriving sentence embeddings and optimizing the models for similarity-based retrieval tasks, would be required to apply domain-specific models to the task explored in the current study. For example, ClinicalBERT produces token-level contextual representations rather than sentence-level embeddings that can be directly compared using cosine similarity to identify semantically similar text. Token-level representations need to be transformed into sentence-level representations using a pooling strategy (eg, mean or max pooling), and different strategies may yield varying retrieval accuracy. Consequently, a meaningful empirical comparison with domain-specific models would be challenging because differences in retrieval accuracy could reflect these adaptations rather than the underlying models themselves. Therefore, rigorous comparison of clinical embedding models was considered beyond the scope of the current exploratory study. Finally, several language categories had very small sample sizes at higher cosine similarity thresholds, resulting in high but statistically unstable precision that should not be misinterpreted as strong or reliable performance.

### Future Research

Future research may consider evaluating recall and *F*_1_-scores, which can provide additional insight into semantic similarity search performance, particularly given the potential trade-off between precision and recall [47]. Exploring additional techniques to refine the candidate selection process may further contribute to optimizing accuracy. For instance, combining other similarity metrics, such as pragmatic similarity, could provide a more comprehensive assessment. Pragmatic similarity considers the intent and attitude behind the text, which could help to ensure that the intended meanings between texts are aligned [48]. In addition, comparing the performance of general-domain and clinical-domain models in similarity-based retrieval tasks for annotation support could provide further insight into how domain-specific models affect retrieval accuracy and annotation efficiency. Future work is also needed to further clarify, refine, and standardize the operationalization of these language categories to improve annotation consistency and reproducibility. Finally, application of this approach across diverse clinical settings and datasets will further inform generalizability.

### Conclusions

This study applies semantic similarity search in the context of stigmatizing language to support annotation workflows. Unlike previous research using uncertainty-based querying or synthetic data augmentation, we used human-annotated true exemplars as queries to directly retrieve additional exemplars from real-world clinical notes to support targeted expansion of context-dependent concepts. Findings demonstrate the potential of semantic similarity search to efficiently extract additional exemplars and increase the volume of training data while reducing annotation burden. By optimizing the cosine similarity thresholds, we may further achieve higher precision. These findings provide a foundation for further refinement and application of this approach in other domains requiring efficient ways to extract additional exemplars to augment NLP training data.

## Supplementary material

10.2196/85088 Multimedia Appendix 1 Supplementary tables presenting detailed category-specific results, synthetic note snippets, and the annotation codebook.

10.2196/85088 Multimedia Appendix 2 Pseudocode for semantic similarity search pipeline.

## Abbreviations

BERT: bidirectional encoder representations from transformers
EHR: electronic health record
IRB: institutional review board
NLP: natural language processing
