# Supplementary material for: Semantic Similarity Search Approach to Extract Exemplars of Stigmatizing and Positive Language in Obstetric Clinical Notes: Exploratory Study
Source: JMIR Med Inform. 2026 Sep 22;14:e85088. doi: 10.2196/85088 (PMC13597023; doi:10.2196/85088)
Supplement: Supplementary file 1 [file medinform-v14-e85088-s001.docx]

**Multimedia Appendix 2. Supplementary Tables**

**Table S1.** Precision at different cosine similarity thresholds by language categories

|  | Cosine Similarity Threshold | | | | | | | | | |
| --- | --- | --- | --- | --- | --- | --- | --- | --- | --- | --- |
|  | 0.50 | | 0.55 | | 0.60 | | 0.65 | | 0.70 | |
| Language Categories | TP/n ^a^ | Precision | TP/n ^a^ | Precision | TP/n ^a^ | Precision | TP/n ^a^ | Precision | TP/n ^a^ | Precision |
| Preferred language | 146/215 | 0.68 | 139/207 | 0.67 | 122/184 | 0.66 | 93/137 | 0.68 | 74/105 | 0.70 |
| Autonomy for birth | 10/20 | 0.50 | 10/20 | 0.50 | 10/18 | 0.56 | 8/14 | 0.57 | 7/9 | 0.78 |
| Difficult patient | 58/129 | 0.45 | 51/113 | 0.45 | 41/88 | 0.47 | 30/64 | 0.47 | 22/42 | 0.52 |
| Unilateral/  authoritarian decisions | 26/65 | 0.40 | 23/46 | 0.50 | 22/42 | 0.52 | 16/27 | 0.59 | 10/17 | 0.59 |
| Power/privilege | 16/35 | 0.46 | 10/20 | 0.50 | 5/8 | 0.63 | 3/3 | 1.00 ^d^ | 2/2 | 1.00 ^d^ |
| Structural/  Interprofessional  hierarchy | 4/10 | 0.40 | 4/10 | 0.40 | 4/9 | 0.44 | 4/5 | 0.80 | 4/5 | 0.80 |
| Marginalized identities | 100/286 | 0.35 | 76/206 | 0.37 | 30/89 | 0.34 | 12/39 | 0.31 | 4/16 | 0.25 |
| Questioning patient credibility | 16/47 | 0.34 | 15/39 | 0.38 | 9/23 | 0.39 | 5/9 | 0.56 | 3/4 | 0.75 ^d^ |
| Disapproval | 7/24 | 0.29 | 5/16 | 0.31 | 2/5 | 0.40 | 2/4 | 0.50 ^d^ | 1/1 | N/R ^c^ |
|  | Cosine Similarity Threshold | | | | | | | | | |
|  | 0.75 | | 0.80 | | 0.85 | | 0.90 | | 0.95 | |
| Language Categories | TP/n ^a^ | Precision | TP/n ^a^ | Precision | TP/n ^a^ | Precision | TP/n ^a^ | Precision | TP/n ^a^ | Precision |
| Preferred language | 41/56 | 0.73 | 20/26 | 0.77 | 12/17 | 0.71 | 6/7 | 0.86 | 2/2 | 1.00 ^d^ |
| Autonomy for birth | 5/5 | 1.00 | 4/4 | 1.00 ^d^ | 2/2 | 1.00 ^d^ | 0/0 | N/A ^b^ | 0/0 | N/A ^b^ |
| Difficult patient | 2/7 | 0.29 | 1/1 | N/R ^c^ | 1/1 | N/R ^c^ | 1/1 | N/R ^c^ | 0/0 | N/A ^b^ |
| Unilateral/  authoritarian decisions | 5/7 | 0.71 | 3/3 | 1.00 ^d^ | 2/2 | 1.00 ^d^ | 1/1 | N/R ^c^ | 0/0 | N/A ^b^ |
| Power/privilege | 1/1 | N/R ^c^ | 1/1 | N/R ^c^ | 1/1 | N/R ^c^ | 1/1 | N/R ^c^ | 0/0 | N/A ^b^ |
| Structural/  Interprofessional  hierarchy | 3/4 | 0.75 ^d^ | 2/2 | 1.00 ^d^ | 2/2 | 1.00 ^d^ | 2/2 | 1.00 ^d^ | 1/1 | N/R ^c^ |
| Marginalized identities | 3/9 | 0.33 | 1/4 | 0.25 ^d^ | 1/1 | N/R ^c^ | 0/0 | N/A ^b^ | 0/0 | N/A ^b^ |
| Questioning patient credibility | 3/3 | 1.00 ^d^ | 3/3 | 1.00 ^d^ | 2/2 | 1.00 ^d^ | 1/1 | N/R ^c^ | 0/0 | N/A ^b^ |
| Disapproval | 1/1 | N/R ^c^ | 0/0 | N/A ^b^ | 0/0 | N/A ^b^ | 0/0 | N/A ^b^ | 0/0 | N/A ^b^ |
| ^a^ n indicates the number of exemplar candidates retained at each cosine similarity threshold, which was used as the denominator to calculate the precision. TP indicates the number of true positives, which was used as the numerator to calculate the precision.  ^b^ N/A (not applicable). Precision could not be calculated when no exemplar candidates were retained at a given threshold (n = 0). Therefore, these instances were reported as not applicable.  ^c^ N/R (not reported). Precision values are mathematically calculable but statistically uninformative when only one exemplar candidate was retained at a given threshold (n = 1). Therefore, these instances were not reported.  ^d^ Precision values based on small sample sizes (1 < n < 5) were calculated and reported but are statistically unstable and should be interpreted with caution. These values should not be interpreted as evidence of strong performance. | | | | | | | | | | |

**Table S2.** Precision at different cosine similarity threshold by language categories with corresponding confidence intervals

|  | **Cosine similarity threshold** | | | | | | | | | | | | | | | | | | | |
| --- | --- | --- | --- | --- | --- | --- | --- | --- | --- | --- | --- | --- | --- | --- | --- | --- | --- | --- | --- | --- |
| **Language categories** | **0.50** | | **0.55** | | **0.60** | | **0.65** | | **0.70** | | **0.75** | | **0.80** | | **0.85** | | **0.90** | | **0.95** | |
|  | **prec** | **CI** | **prec** | **CI** | **prec** | **CI** | **prec** | **CI** | **prec** | **CI** | **prec** | **CI** | **prec** | **CI** | **prec** | **CI** | **prec** | **CI** | **prec** | **CI** |
| Preferred language | 0.68 | 0.61-0.74 | 0.67 | 0.60-0.73 | 0.66 | 0.59-0.73 | 0.68 | 0.60-0.75 | **0.70** | 0.61-0.78 | **0.73** | 0.60-0.83 | **0.77** | 0.58-0.89 | **0.71** | 0.47-0.87 | **0.86** | 0.49-0.97 | **1.00** | 0.34-1.00 |
| Autonomy for birth | 0.50 | 0.30-0.70 | 0.50 | 0.30-0.70 | 0.56 | 0.34-0.75 | 0.57 | 0.33-0.79 | **0.78** | 0.45-0.94 | **1.00** | 0.57-1.00 | **1.00** | 0.51-1.00 | **1.00** | 0.34-1.00 | n/a | n/a | n/a | n/a |
| Difficult patient | 0.45 | 0.37-0.54 | 0.45 | 0.36-0.54 | 0.47 | 0.37-0.57 | 0.47 | 0.35-0.59 | 0.52 | 0.38-0.67 | 0.29 | 0.08-0.64 | **1.00** | 0.21-1.00 | **1.00** | 0.21-1.00 | **1.00** | 0.21-1.00 | n/a | n/a |
| Unilateral/  authoritarian decisions | 0.40 | 0.29-0.52 | 0.50 | 0.36-0.64 | 0.52 | 0.38-0.67 | 0.59 | 0.41-0.75 | 0.59 | 0.36-0.78 | **0.71** | 0.36-0.92 | **1.00** | 0.44-1.00 | **1.00** | 0.34-1.00 | **1.00** | 0.21-1.00 | n/a | n/a |
| Power/privilege | 0.46 | 0.30-0.62 | 0.50 | 0.30-0.70 | 0.63 | 0.31-0.86 | **1.00** | 0.44-1.00 | **1.00** | 0.34-1.00 | **1.00** | 0.21-1.00 | **1.00** | 0.21-1.00 | **1.00** | 0.21-1.00 | **1.00** | 0.21-1.00 | n/a | n/a |
| Structural/  Interprofessional  hierarchy | 0.40 | 0.17-0.69 | 0.40 | 0.17-0.69 | 0.44 | 0.19-0.73 | **0.80** | 0.38-0.96 | **0.80** | 0.38-0.96 | **0.75** | 0.30-0.95 | **1.00** | 0.34-1.00 | **1.00** | 0.34-1.00 | **1.00** | 0.34-1.00 | **1.00** | 0.21-1.00 |
| Marginalized identities | 0.35 | 0.30-0.41 | 0.37 | 0.31-0.44 | 0.34 | 0.25-0.44 | 0.31 | 0.19-0.46 | 0.25 | 0.10-0.49 | 0.33 | 0.12-0.65 | 0.25 | 0.05-0.70 | **1.00** | 0.21-1.00 | n/a | n/a | n/a | n/a |
| Questioning patient credibility | 0.34 | 0.22-0.48 | 0.38 | 0.25-0.54 | 0.39 | 0.22-0.59 | 0.56 | 0.27-0.81 | **0.75** | 0.30-0.95 | **1.00** | 0.44-1.00 | **1.00** | 0.44-1.00 | **1.00** | 0.34-1.00 | **1.00** | 0.21-1.00 | n/a | n/a |
| Disapproval | 0.29 | 0.15-0.49 | 0.31 | 0.14-0.56 | 0.40 | 0.12-0.77 | 0.50 | 0.15-0.85 | **1.00** | 0.21-1.00 | **1.00** | 0.21-1.00 | n/a | n/a | n/a | n/a | n/a | n/a | n/a | n/a |

*Note.* Bolded precision values represents acceptable range (≥ 0.7). Precision could not be calculated when no exemplar candidates were retained at a given threshold; thus, these instances were reported as not applicable.

Abbreviations: prec=precision, n/a=not applicable, CI=confidence interval

**Table S3.** Examples of synthetic, free-text note snippets

Pt presents for routine PNV at 24 wks GA. Denies ctx, VB, LOF. +FM. Reviewed routine precautions.

PPD#1 s/p C/S. Pain controlled w PO meds. Ambulating and tolerating diet. Incision c/d/i.

G3P2 at 38+4 wks presents to triage c/o lower abd cramping since last night. No cervical change. NST reactive. D/c home w return precautions.

Pt w h/o OUD on MAT. Reports compliance w meds and f/u. Discussed postpartum plan and support resources.

PPD#2 s/p SVD. Pain well controlled w PO meds. Lochia appropriate. Bonding well w infant.

Pt w h/o SUD, limited PNC and missed prior appts. States trying to stay engaged in care. SW consulted.

Pt reports active engagement in tx program and adherence to MAT. Team acknowledged efforts and discussed continued support during pregnancy.

Pt admitted for IOL d/t gHTN. BP stable overnight. No HA, vision changes, or RUQ pain.

PNV notable for elevated BP x2. Pt denies HA, vision changes, or RUQ pain. Plan for repeat BP and close f/u.

G2P1 at 39+1 wks presents w ctx q5–7 min. Cervix unchanged from prior exam. Pt reassured and d/c home.

**Table S4.** Category codebook for qualitative annotation.

| **Domain** | **Category** | **Definition** | **Common Words, Expressions, Phrases** | **Examples** | **Inclusion Criteria** | **Exclusion Criteria** |
| --- | --- | --- | --- | --- | --- | --- |
| Stigmatizing, toward patients | Difficult patient | Nonadherence or noncompliance with plan of care, refusal of care referrals or services. | Noncompliant, complains, poor compliance, refuses, difficult, argumentative, uncooperative, will not follow instructions, explained multiple times, despite explanation | “missed nutrition appt again says she doesn’t want to go”  “exam unchanged however now c/o [complains of] ctx [contraction] pain”  “’poor compliance with antenatal visits” | Statements portraying the patient as difficult, resistant, uncooperative, or problematic. | Neutral, objective documentation of clinical signs or symptom reports without value-laden statements. |
| Stigmatizing, toward patients | Unilateral/  authoritarian decisions | Language that supports clinician's authority over patients. Upholds hierarchy centering clinician, not patient. | Told to do, instructed to, if then consequences (authorities will need to be contacted), comply, required to, non-negotiable, mandated | “cervix unfavorable therefore will start induction with cytotec”  “SW [social worker] has advised pt [patient] that if there continues to be yelling in room, ACS [adult and child services] will need to be contacted”  “also very upset about persistent shaking in her UE [upper extremities]. Explained to patient multiple times that shaking is normal and a result of hormones and the anesthesia” | Statements that emphasize authority, unilateral decision-making, or consequences, clinician control rather than patient preference. | - Statements conveying recommended actions based on clinical judgment within shared decision-making. - Neutral descriptions of clinical plans or treatment without authority or control statements. |
| Stigmatizing, toward patients | Power/privilege | Describes power and privilege identities related to psychological or social-ecological status. | private patient, works as a high-status profession, well-educated, from good family, well-groomed or looking, nurturing marriage (when emphasized as status marker), Caucasian (when not clinically relevant), pleasant or articulate (when linked to social valuation), stable home, high-functioning | “pt [patient] reports having a nurturing marriage with FOB [father of the baby] who works as a Lawyer”  “private pt of mine at 39+6 wks [weeks] with multiple episodes of emesis this AM”  “Caucasian female. pleasantly engaged in conversation” | - Statements that emphasize social identities in ways that signal status, privilege, credibility without direct clinical relevance. - Statements that indicate favorable social status. | Documentation or assessment for information that are clinically relevant and/or as a part of structured fields (no free-text). |
| Stigmatizing, toward patients | Marginalized identities | Documentation of social and behavioral risk factors in narrative that could contribute to marginalization.  Restating for emphasis that is already in the checklist/form data or unnecessary patient descriptor (e.g., “toxic habits,” "financially supports self," “teen mother,”  “late registrant,” and “obesity”). | Quoting incorrect grammar or unsophisticated terms, quoting vernacular English, teen mother, toxic habits, late registrant, financially supports self (when irrelevant to care), labeling and stereotyping terms | “social disarray, estranged from family with FOB [father of the baby] not involved”  “Dominican female. Initial psychosocial assessment due to teen pregnancy late registrant”  “Patient is a 32yo [year old] Dominican unmarried unemployed female” | - Statements that convey an opinion about a patient based on preconceived ideas about their culture may be unjustified. - Statements indicating assessment of social and behavioral risks that may be unjustified. - Restating social risk factors already documented in structured fields. - Emphasis on race and ethnicity without clinical relevance. | - Neutral, objective statements of clinically relevant a patient's culture (e.g., the patient does not accept blood products because they are Jehovah's witness). - Documentation or assessment for information that are clinically relevant and/or as a part of structured fields (no free-text). |
| Stigmatizing, toward patients | Questioning patient credibility | Disbelief in patient’s report of health and social history or status. | inconsistent history, poor history, story does not add up, denies despite evidence or records, claims, doubtful, skeptical, unlikely explanation | “unsure if patient telling the truth”  “pt [patient] denied any other DV [domestic violence] incidents and was adamant that relationship with spouse was healthy.”  “Pt does not think she has gestational diabetes she states she ate very sweet rice (a dish from her country) the day before the glucose challenge which is what she believes this is the reason for the high value states it has never been high before.” | - Statements that question the accuracy, truthfulness, or reliability of the patient’s self-report, such as using quotations. - Language that conveys doubt, skepticism, or disbelief toward the patient. | - Objective clinical assessments (e.g., laboratory results) to support statements. - Quotation is used to neutrally and objectively describe patient reports. |
| Stigmatizing, toward patients | Disapproval | Behaviors of patient not in alignment with healthcare provider expectations. | Adamant, despite talking extensively, despite extensive counseling, despite importance stressed, repeatedly advised, inappropriate decision or behavior, poor judgment, will continue to readdress | “FOB [father of baby] is 23 yo [year old] unemployed and not as involved as he should be.”  “Pt [patient] states she tried to obtain f/u [follow up] appt [appointment]. but did not get a call back. Advised she should have gone to the clinic to straighten things out- in the future to take initiative-importance stressed as well as appt.s for her newborn”  “PP [postpartum] BCM [bridge contraceptive method]- pt states she prefers to use condoms will continue to readdress” | Statements that convey criticism, disapproval, or moral judgment toward a patient’s decisions, reasoning, or self-care behaviors, particularly when language implies that the patient failed to meet or contradicts provider expectations or values. | Objective statements noting that a patient has decided not to receive treatment (e.g., patient declines). |
| Stigmatizing, among clinicians, adverse impact on care for patients | Structural/ interprofessional hierarchy | Notes stating issues in care are attributed to another clinician, often nurses. Notes stating that care was not delivered in a timely manner due to staffing or other structural issues. | nurse states, per nursing, unable to staff, staff unavailable, waiting on nursing, delay due to staffing, not done by prior team | “Nurse states IV [intravenous] ‘infiltrated’, but it was fine.”  “Unable to staff nursing for placement of epidural in triage. Requested that charge nurse please inform anesthesiology team about when there is enough nursing staff.” “Patient continues to wait in triage until L D [Labor & Delivery] bed available.” | Statements that attribute delays, errors, or inadequacy in care to another health care team member or group in ways that imply blame, distancing, or hierarchical positioning, particularly when framed as justification for suboptimal care. | - Neutral documentation of staffing limitations, bed availability, or workflow constraints when presented without blaming tone or language. - Routine communication notes (e.g., notified anesthesia). |
| Positive (non-stigmatizing), for patients | Preferred language | Preferred words that can convey patient’s point of view respectively and objectively (e.g., endorses, reports, or states). | Endorses, reports, states, desires, expresses, describes, prefers, requests | “patient desires to ambulate and encourage natural labor.”  “patient states she feels some pain on right side.”  “states she has had irregular ctx [contractions] starting 24 hours ago. endorses FM [fetal movement]. Endorses N/V [nausea/vomiting]” | Statements that document the patient’s symptoms, experiences, or preferences using neutral, respectful, and patient-centered language that clearly attributes the information to the patient without judgment. | Statements of objective clinical findings assessments (e.g., laboratory results, exam findings) that do not involve patient reports, experiences, or preferences. |
| Positive (non-stigmatizing), for patients | Autonomy for birth | Notes that indicate patient exercising autonomy around birth and plan of care. | Declines, offer options, give options, patient decides, chooses, | “Pt [patient] declines epidural at this time- trying to deliver without analgesia.”  “will give patient the option to have continuous monitoring overnight and give her the opportunity to go in to spontaneous labor with plans for c-section tomorrow if she does not progress.”  “low risk screening declined diagnostic. She was given the option of admission with possible augmentation of labor vs. discharge home to labor. Plan was made for admission.” | Statements that document patient choice, preference, or participation in care decisions, including shared decision-making, without judgmental, corrective, or coercive language. | Statements of treatment plan without reference to patient participation or choice.  Statements of patient refusal or decline with judgmental, corrective, or coercive language. |

*Note.* The presence of common words, expressions, and phrases alone does not indicate automatic inclusion. Similarly, absence of common words, expressions, and phrases alone does not indicate automatic exclusion. Annotators must review the full free-text narratives and evaluate contextual elements (e.g., clinical situations, tone, framing).
