# Supplementary material for: Semantic Similarity Search Approach to Extract Exemplars of Stigmatizing and Positive Language in Obstetric Clinical Notes: Exploratory Study
Source: JMIR Med Inform. 2026 Sep 22;14:e85088. doi: 10.2196/85088 (PMC13597023; doi:10.2196/85088)
Supplement: Supplementary file 2 [file medinform-v14-e85088-s002.docx]

**Multimedia Appendix 1. Pseudo-Code for Semantic Similarity Search Pipeline**

The following pseudo-code describes the end-to-end pipeline used to retrieve exemplar candidates of stigmatizing and positive language from obstetric clinical notes. The pipeline was implemented in Python 3. Key packages and their roles are listed below, followed by a step-by-step algorithmic description.

**Software and Packages**

| **Component** | **Package / Tool** |
| --- | --- |
| Language | Python 3 |
| Sentence embedding | sentence-transformers (Reimers & Gurevych, 2019) |
| Pretrained model | multi-qa-distilbert-cos-v1 (Hugging Face Hub) |
| Similarity search | faiss-cpu (Johnson et al., 2017) |
| Sentence splitting | nltk.tokenize.sent_tokenize (NLTK) |
| Data handling | pandas, numpy |

**Algorithm 1: Semantic Similarity Search for Exemplar Extraction**

**INPUT**

N = Clinical notes from labor/birth admissions (7 note types)

Q = 200 true exemplars, stratified by language category

k = 5 (top-k candidates per query)

model = "multi-qa-distilbert-cos-v1"

Θ = Cosine similarity thresholds {0.50, 0.55, …, 0.95}

**OUTPUT**

Exemplar candidates with similarity scores, reviewed by annotators

Precision at each threshold Θ with 95% Wilson confidence intervals

**STEP 1: SENTENCE WINDOW PREPARATION**

```
FOR each note n in N (excluding notes used in initial annotation):
    sentences ← nltk.sent_tokenize(n)
    FOR window_size in {1, 2, 3}:
        FOR i = 0 TO len(sentences) - window_size:
            w ← join(sentences[i], …, sentences[i + window_size - 1])
            ADD w to candidate corpus W
        END FOR
    END FOR
END FOR

// Deduplication (two-stage, six-word prefix matching)
FOR each window w in W:
    prefix ← first 6 words of w
    IF prefix already seen: MARK w as duplicate
END FOR
REMOVE duplicates, keeping earliest instance
REPEAT pass to remove any remaining duplicates

// No text normalization applied; original casing, numerics,
// and abbreviations preserved to maintain clinical text fidelity
```

**STEP 2: EMBEDDING AND INDEXING**

```
model ← SentenceTransformer("multi-qa-distilbert-cos-v1")

// Encode all candidate windows
E_W ← model.encode(W, normalize_embeddings=True)
    // Output: matrix of L2-normalized vectors (dim = 768)

// Build FAISS index for cosine similarity search
// (inner product on L2-normalized vectors = cosine similarity)
index ← faiss.IndexFlatIP(dimension=768)
index.add(E_W)
```

**STEP 3: QUERY AND RETRIEVAL**

```
// Stratified selection of 200 true exemplars as queries
Q ← stratified_random_sample(true_exemplars, n=200,
        stratify_by=language_category)

// Encode queries and retrieve top-k candidates
E_Q ← model.encode(Q, normalize_embeddings=True)
scores, indices ← index.search(E_Q, k=5)

// Compile results
candidates ← empty list
FOR each query q_i in Q:
    FOR rank = 1 TO k:
        RECORD {query_text: q_i,
                candidate_text: W[indices[i][rank]],
                similarity_score: scores[i][rank],
                rank: rank}
    END FOR
END FOR

// Deduplicate across queries (retain highest similarity score)
candidates ← sort by similarity_score descending
candidates ← drop duplicates on candidate_text, keep first

// Total candidates retrieved: N = 1,000
```

**STEP 4: PRECISION EVALUATION**

```
// Precision at each cosine similarity threshold
FOR each threshold t in {0.50, 0.55, …, 0.95}:
    filtered ← candidates WHERE similarity_score ≥ t
    n_total  ← count(filtered)
    n_match  ← count(filtered WHERE final_label = "Match")
    IF n_total > 0:
        precision(t) ← n_match / n_total
        CI(t) ← Wilson_confidence_interval(n_match, n_total, α=0.05)
    ELSE:
        precision(t) ← N/A
END FOR

// Category-level precision
FOR each language_category:
    REPEAT precision calculation within category subset
END FOR
```
